# The Mouse Universal Genotyping Array: From Substrains to Subspecies

**DOI:** 10.1534/g3.115.022087

**Published:** 2015-12-18

**Authors:** Andrew P. Morgan, Chen-Ping Fu, Chia-Yu Kao, Catherine E. Welsh, John P. Didion, Liran Yadgary, Leeanna Hyacinth, Martin T. Ferris, Timothy A. Bell, Darla R. Miller, Paola Giusti-Rodriguez, Randal J. Nonneman, Kevin D. Cook, Jason K. Whitmire, Lisa E. Gralinski, Mark Keller, Alan D. Attie, Gary A. Churchill, Petko Petkov, Patrick F. Sullivan, Jennifer R. Brennan, Leonard McMillan, Fernando Pardo-Manuel de Villena

**Affiliations:** * Department of Genetics, University of North Carolina, Chapel Hill, North Carolina 27599; † Lineberger Comprehensive Cancer Center and Carolina Center for Genome Sciences, University of North Carolina, Chapel Hill, North Carolina 27599; ‡ Department of Computer Science, University of North Carolina, Chapel Hill, North Carolina 27599; § Department of Mathematics and Computer Science, Rhodes College, Memphis, Tennessee 38112; ** Department of Epidemiology, University of North Carolina, Chapel Hill, North Carolina 27599; §§ Department of Psychiatry, University of North Carolina, Chapel Hill, North Carolina 27599; †† Department of Biochemistry, University of Wisconsin, Madison, Wisconsin 53706; ‡‡ The Jackson Laboratory, Bar Harbor, Maine 04609; *** Mutant Mouse Resource and Research Center, University of North Carolina, Chapel Hill, North Carolina 27599

**Keywords:** microarrays, genetic mapping, inbred strains

## Abstract

Genotyping microarrays are an important resource for genetic mapping, population genetics, and monitoring of the genetic integrity of laboratory stocks. We have developed the third generation of the Mouse Universal Genotyping Array (MUGA) series, GigaMUGA, a 143,259-probe Illumina Infinium II array for the house mouse (*Mus musculus*). The bulk of the content of GigaMUGA is optimized for genetic mapping in the Collaborative Cross and Diversity Outbred populations, and for substrain-level identification of laboratory mice. In addition to 141,090 single nucleotide polymorphism probes, GigaMUGA contains 2006 probes for copy number concentrated in structurally polymorphic regions of the mouse genome. The performance of the array is characterized in a set of 500 high-quality reference samples spanning laboratory inbred strains, recombinant inbred lines, outbred stocks, and wild-caught mice. GigaMUGA is highly informative across a wide range of genetically diverse samples, from laboratory substrains to other *Mus* species. In addition to describing the content and performance of the array, we provide detailed probe-level annotation and recommendations for quality control.

High-throughput genotyping of single nucleotide polymorphisms (SNPs) using oligonucleotide microarrays is now standard practice in genetics. SNPs have largely supplanted microsatellite loci as the markers of choice for genome-wide genotyping: the low information content of individual (biallelic) SNP markers relative to (multiallelic) microsatellites is overcome by the ability to simultaneously type many thousands of SNPs (The International HapMap Consortium 2005). Current technologies provide rapid, robust, and accurate genotyping of hundreds of thousands of markers at a cost of less than $0.001 per genotype.

Unlike sequencing approaches, which ascertain and genotype polymorphic sites in the study population in a single pass, arrays interrogate a fixed number of known sites. This presents an optimization problem: given a set of known SNPs, what subset provides maximal information content for the populations and experiments of interest? Marker selection also raises the possibility of ascertainment bias (Clark *et al.* 2005). In this manuscript, we describe the Mouse Universal Genotyping Array (MUGA), a general-purpose genotyping array for the laboratory mouse (*Mus musculus*), and discuss the strategies used for SNP selection with respect to global and local information content.

The first mouse genotyping arrays were based on polymorphism data from a limited number of laboratory strains (Lindblad-Toh *et al.* 2000; Shifman *et al.* 2006). Their content was biased heavily toward alleles segregating in the subspecies *Mus musculus domesticus*, the predominant ancestral component of classical laboratory mice (Yang *et al.* 2007). Next, the Mouse Diversity Array (MDA) was designed to interrogate variation across a broader swath of the mouse phylogeny (Yang *et al.* 2009), taking advantage of new sources of polymorphism data (Frazer *et al.* 2007). The MDA enabled characterization of the ancestry of laboratory strains and wild mice (Yang *et al.* 2011), construction of high-resolution recombination maps (Liu *et al.* 2014), and haplotype inference in recombinant inbred panels including the Collaborative Cross (Aylor *et al.* 2011). However, the MDA is relatively expensive for routine use and its sample-preparation procedure is labor-intensive.

The MUGA was designed to fill a need for a low-cost (approximately $100 per sample) genotyping platform to support the development of the Collaborative Cross (CC) (Collaborative Cross Consortium 2012; Welsh *et al.* 2012), and Diversity Outbred (DO) (Svenson *et al.* 2012) populations . MUGA was developed on the Illumina Infinium platform (Steemers *et al.* 2006), in cooperation with Neogen Inc. (Lincoln, NE). The 7851 SNP markers on the first-generation MUGA were spaced uniformly every ∼325 kb across the mouse reference genome and were selected to uniquely identify the eight founder haplotypes of the CC and DO—A/J, C57BL/6J, 129S1/SvImJ, NOD/ShiLtJ, NZO/HlLtJ, CAST/EiJ, PWK/PhJ and WSB/EiJ—in any window of 3−5 consecutive markers. Although MUGA was reliable and inexpensive, it lacked the marker density to capture the increasing number of recombination events in later generations of the DO (Churchill *et al.* 2012). It provided less phylogenetic coverage, and limited discrimination between closely related laboratory strains in comparison to the MDA, and had narrower dynamic range, making it less useful for copy-number analyses. The second-generation MegaMUGA, available in 2012, was designed to address some of these limitations. It provided 10-fold greater marker density than the first-generation MUGA (77,808 markers), again mostly optimized for information content in the CC and DO (about 65,000 markers), but with an additional 14,000 probes targeting variants segregating in wild-caught mice and wild-derived strains. The remaining fraction of the array (about 1000 markers) included markers segregating between C57BL/6J and C57BL/6NJ, and probes targeted to transgenes and other engineered constructs (Morgan and Welsh 2015). In contrast to MUGA, the content of MegaMUGA was optimized for discriminating between CC founder haplotypes in both homozygous and heterozygous states.

The MUGA and MegaMUGA arrays have been used for monitoring of inbreeding in the CC (Collaborative Cross Consortium 2012), and for quantitative-trait mapping in outbred stocks (Svenson *et al.* 2012; Gatti *et al.* 2014) and experimental crosses (Rogala *et al.* 2014; Carbonetto *et al.* 2014). They have also been deployed to detect contamination and aneuploidy in cell lines (Didion *et al.* 2014), and to characterize structural variants in inbred lines (Calaway *et al.* 2013; Crowley *et al.* 2015; Didion *et al.* 2015).

GigaMUGA, the third generation in the MUGA family, improves on MegaMUGA by providing a further increase in marker density (to 143,259 markers) and substantially expanded content. The design goals of GigaMUGA were fourfold: (1) to increase resolution for detecting recombination events in the CC and DO; (2) to increase power to discriminate between closely related laboratory strains; (3) to increase information content for wild-caught mice and wild-derived lines; and (4) to assay copy number in genomic regions prone to structural variation. Approximately half of the array is comprised of validated CC-/DO-targeted markers carried over from MegaMUGA. An additional set of 46,000 markers flank recombination hotspots predicted to be active in the CC and DO (Baker *et al.* 2015). About 15,000 probes target SNPs ascertained in widely used laboratory mice, including the 129, BALB, C3H, C57BL/6, and DBA strain complexes, and the ICR outbred stock. Another 7700 probes were designed against SNPs segregating in wild mice of *M. m. domesticus, M. m. musculus* and *M. m. castaneus* ancestry. Finally, 2000 probes were spaced across segmental duplications to detect copy-number variation (CNV) in these mutation-prone regions of the genome (Egan *et al.* 2007).

In this paper, we describe the selection of markers for the GigaMUGA platform and characterize their performance in a set of 500 reference samples spanning classical laboratory strains, wild-derived strains, wild-caught mice, and sister species from the *Mus* genus. We highlight the utility of GigaMUGA for substrain-level identification of laboratory mice.

## Materials and Methods

### Microarray platform

GigaMUGA was designed on the Illumina Infinium HD platform (Steemers *et al.* 2006). Invariable oligonucleotide probes 50 bp in length are conjugated to silica beads that are then addressed to wells on a chip. Sample DNA is hybridized to the oligonucleotide probes and a single-base-pair templated-extension reaction is performed with fluorescently labeled nucleotides. Nucleotides are labeled such that one bead is required to genotype most SNPs, and two beads for [A/T] and [C/G] SNPs. The relative signal intensity from alternate fluorophores at the target nucleotide is processed into a discrete genotype call (AA, AB, BB) using the Illumina BeadStudio software. Although the two-color Infinium readout is optimized for genotyping biallelic SNPs, both total and relative signal intensity are also informative for copy-number changes.

### Probe design

The vast majority of probes (141,090; 98.5%) on GigaMUGA target biallelic SNPs. The remaining 2169 probes fall in two classes. The first class consists of presence-absence probes for engineered constructs or known structural variants (*e.g.*, *Mx1*, *R2d2*). The second class consists of copy-number probes. In order to maximize usage of space the array, target SNPs were biased toward (single-bead) transitions (final transition:transversion ratio = 3.83).

#### Informative SNPs in the CC and DO populations:

The bulk of the content of GigaMUGA was designed to interrogate SNPs segregating in the eight CC and DO founder strains ascertained by the Sanger Mouse Genomes Project (Keane *et al.* 2011), and the MDA. The subset of SNPs targeted by GigaMUGA was selected to maximize discrimination between the eight homozygous CC founder haplotypes as well as their $\left(\begin{array}{c} 8\\ 2 \end{array}\right)=28$ possible heterozygous combinations (ignoring phase.) First, candidate target SNPs were identified as SNPs assayable with a single bead, located at least 50 bp from any adjacent SNP or indel, and whose 50-bp flanking sequences are unique in the reference genome. Each chromosome was then divided into *n* target intervals of uniform size on the genetic map (Liu *et al.* 2014) such that each interval contained at least one candidate target SNP.

One target SNP was chosen per target interval using a dynamic-programming-like algorithm as follows. Define a path (*q*) as a sequence of one target SNP per target interval along a chromosome. Possible paths were scored via a score function f(.) by counting the total number (1≤k≤36) of genotype states that can be distinguished in five-SNP sliding windows along the path; denote the score on path *q* for the first *i* intervals S(i,q). Although the number of possible paths is exponential in the number of target intervals, the score follows the recurrence relation:

$$S\left(i+1,q+s\right)=S\left(i,q\right)+\underset{s\in V}{\mathrm{argmax}}f\left(q_{-4}+s\right)$$

where *s* is a candidate target SNP; *V* is the set of candidate target SNPs in interval i+1; $q_{-4}$ is the last four SNPs along the current path; and f(.) is the scoring function for a single five-SNP window.

Scores for possible paths along each chromosome were calculated, pruning the set of paths to keep only the highest-scoring $1\times 10^{5}$ paths at each step. The (approximately) optimal set of SNPs for each chromosome was then chosen by tracing back along the path with maximum S(n,q). A total of 54,250 probes was selected in this manner, all carried over from the MegaMUGA array. The majority (53,529) were selected from the Sanger Mouse Genomes Project SNP calls; 666 were carried over from the MDA.

An additional 46,020 probes were designed to target SNPs flanking 25,000 predicted recombination hotspots associated with *Prdm9* alleles segregating in the CC and DO (Baker *et al.* 2015). These SNPs were selected to be locally informative in four-SNP windows overlapping the central 100 bp of each *Prdm9* binding site, so instead of using the recursion introduced above, we selected SNPs maximizing the local scoring function f(.) around each hotspot rather than along entire chromosomes.

Finally, to fill any remaining gaps, probes were designed against a further 1,943 Sanger SNPs predicted to be segregating in the CC and DO.

#### Informative SNPs in common laboratory mouse strains:

To boost informativeness of the array for laboratory stocks not represented in the Sanger Mouse Genomes Project, we included SNPs from two sources: MDA, and resequencing of selection lines derived from a common outbred stock. First, 13,036 additional MDA probes informative among laboratory mice were carried over to GigaMUGA.

Second, SNPs were ascertained from whole-genome sequencing (20−30×) of five selection lines (the “high-runner” or HR lines) derived from the ICR:Hsd outbred stock (Swallow *et al.* 1998). This stock has a similar genetic background to a group of commonly used laboratory strains (so-called “Swiss mice”) (Beck *et al.* 2000). Briefly, reads from one individual from each of the five selection lines were aligned to the mouse reference genome (mm9/GRCm37 build) using bowtie2 v2.2.3 (Langmead and Salzberg 2012) with default options. Suspected PCR duplicates were removed using Picard v1.88 (http://picard.sourceforge.net/). SNPs were called using samtools mpileup v0.1.19-44428cd (Li *et al.* 2009) and filtered against the Sanger Mouse Genomes Project variant catalog. We targeted the resulting novel SNPs for inclusion on GigaMUGA if they met several additional criteria: not present on the MegaMUGA array, polymorphic in the five HR samples, and located in regions of low marker density on MegaMUGA array, but high SNP density in the five HR samples. A total of 3693 SNPs from the HR lines was included on the final array.

#### Informative SNPs between closely related strains:

To increase the value of GigaMUGA as a tool for discriminating between closely related inbred strains, we used data from MegaMUGA, MDA, and the Sanger Mouse Genomes Project to identify variants segregating between substrains. We included all 139 MegaMUGA probes discriminating between substrains of C57BL/6, and designed probes for an additional 251 variants between C57BL/6J and C57BL/6NJ ascertained by the Sanger Mouse Genomes Project. MDA data were used to select 540 variants useful for discriminating between several other substrain pairs: 129S1/SvImJ *vs.* 129S6/SvEvTac (221), A/J *vs.* A/WySnJ (148), AEJ/GnLeJ *vs.* AEJ/GnRk (31), BALB/cJ *vs.* BALB/cByJ (105), C3H/HeJ *vs.* C3HeB/FeJ (96), DBA/1J *vs.* DBA/1LacJ (20), DBA/2J *vs.* DBA/2DeJ (161), SEC/1GnLeJ *vs.* SEC/1ReJ (13), and SJL/Bm *vs.* SJL/J (8). These markers were selected to cover the genome uniformly. In some genomic regions for some strain pairs, many additional markers will be informative. Variation in these regions is not due to mutation and drift since the establishment of the lines, but was either segregating in the ancestors of the inbred line, or is due to contamination from other laboratory stocks.

#### Informative SNPs in wild mice:

To facilitate studies of wild mice, we included SNPs informative for subspecies of origin. Our goal was to achieve a density of at least one “diagnostic marker” per 300 kb for each subspecies, and to place at least one diagnostic marker for each subspecies within each recombination of the intervals identified in Liu *et al.* (2014). We identified diagnostic markers based on a cohort of wild mice genotyped on the MDA (J. P. Didion, unpublished data) using the method of Yang *et al.* (2011). We used a hidden Markov model (HMM) to assign each region of the genome within each individual to one of the three *M. musculus* subspecies using a panel of reference samples of known pure ancestry. We then computed the allele frequency at each MDA marker within each subspecies. Every marker with an allele exclusive to a single subspecies (allowing up to two mismatches) was considered diagnostic for that subspecies.

We next identified regions of the genome in which marker density was lower than 1/300 kb. Within each region, and within each subspecies having less than the required marker density, we performed an iterative search for diagnostic markers using a progressively decreasing minor-allele frequency (MAF) threshold (from 0.45 to 0.00 in steps of 0.05). At each step, we identified all markers with a MAF greater than the threshold, and with as uniform spacing as possible. We next identified recombination intervals that still lacked at least one diagnostic marker for each subspecies, and attempted to select a diagnostic marker at random, if one was available. A total of 12,489 MDA probes was selected for GigaMUGA using this scheme.

In addition, we designed probes for 7748 SNPs ascertained by whole-genome sequencing of two wild-caught *M. m. domesticus* mice (one from eastern Spain, and one from northern Italy), and two wild-derived inbred strains of *M. m. domesticus* ancestry (ZALENDE/EiJ and LEWES/EiJ). Our goal was to identify SNPs in these mice that had not been discovered in the 18 strains sequenced as part of the Sanger Mouse Genomes Project. Briefly, reads were aligned to the mm9 (GRCm37) reference genome using bwa 0.6.2-r126 (Li and Durbin 2009), and local realignment around indels was performed with the Genome Analysis Toolkit (GATK) IndelRealigner v2.4-7-g5e89f01 (McKenna *et al.* 2010). SNPs were called using samtools mpileup v0.1.19-44428cd, and putative variants were filtered (by position only) against dbSNP and the Mouse Genomes Project variant catalog. We then attempted to place three novel SNPs within each 1-Mb window along the genome (two transitions and one transversion), selected at random from all the novel SNPs in that region. We attempted to space them evenly by placing one transition each in the first and second 500-kb windows of each 1-Mb region, and the transversion within the middle 333 kb. We favored SNPs with higher MAF within the four wild mice. We avoided placing SNPs closer than 100 kb apart unless that was the only option for the 1-Mb window.

#### Copy-number probes:

Copy-number variants in laboratory mouse strains are clustered near tracts of large (>10 kb) tandem segmental duplications (SDs) (She *et al.* 2008). Several groups, including ours, have recognized that SDs are a source of recurrent *de novo* structural variation in mouse (Egan *et al.* 2007; Liu *et al.* 2014). Most SD-rich regions of the mouse genome are also “cold regions” for meiotic recombination, and we have hypothesized that these patterns are causally related (Liu *et al.* 2014).

Although not optimized for detecting copy-number changes in the same manner as tiling arrays (aCGH), hybridization intensity on SNP arrays can capture the signal of aberrant copy number. Increased (or decreased) copy number of a genomic region should result in higher (lower) hybridization intensity at SNPs within the region. Although signal from a single SNP probe is noisy [and may be confounded by off-target variation in or near the probe sequence (Didion *et al.* 2012)], the aggregate signal across many consecutive probes is informative (see, for example, Crowley *et al.* 2015; Didion *et al.* 2015).

We designed a subset of 2006 probes to detect CNVs in 22 SD-rich cold regions described in Liu *et al.* (2014). First the genomic sequence (from the mm10/GRCm38 reference assembly) for each of 59 target regions was extracted and aligned to itself using lastz (http://www.bx.psu.edu/~rsharris/lastz/). Segmentally duplicated intervals were identified as intervals of self-similarity (>95%) longer than 10 kb. Every such interval is, by definition, present more than once; we retained the interval with the smallest genomic coordinate as the unique representative of that sequence. Because the Illumina postprocessing software is optimized for probes with signal from two alleles (an *x*- and *y*-coordinate), we next identified paralogous SNPs (positions that vary between copies of a duplicated sequence on the same chromosome) within the duplicated intervals. Using samtools mpileup on BAM files from the Sanger Mouse Genomes Project, we identified paralogous SNPs as any positions with evidence for both pseudoheterozygosity (>3 reads containing each of two or more bases), and excess coverage (read depth >50). Probe sequences were designed as 50-mers extending upstream from (or downstream from the reverse complement of) each paralogous SNP located >50 bp away from another paralogous SNP. Of 2338 such candidate probes, 2006 were successfully fabricated on the array.

#### Probes for complement cascade genes:

Putative functional SNPs in 25 genes in the complement cascade (Table 5) were targeted as follows. First we identified biallelic variants in transcribed regions of the 25 target genes that are segregating in the eight founder strains of the CC using data from the Sanger Mouse Genomes Project. Variants within 50 bp of another variant were filtered. Probes were designed against the resulting 803 variants; of these, 105 were included on the final array.

#### Probes for genetically engineered constructs:

To increase the utility of GigaMUGA for verifying the integrity of genetically engineered mice, a set of 87 probes was carried over from the MegaMUGA array. These were designed to assay the presence or absence of a variety of transgenes and other exogenous constructs including the Cre and iCre recombinases; reporters such as LacZ and GFP; the CMV, SV40, and rabbit β-globin promoter sequences; and resistance cassettes to tetracycline, chloramphenicol, neomycin, puromycin, hygromycin, and ampicillin. Probe sequences were designed against 51 bp of known construct sequence; alternate alleles were arbitrarily selected and are not informative. Of this group of probes, 79 were successfully fabricated on the final array.

#### Genomic annotation:

Genomic positions were assigned for all markers on the array by mapping the final manufactured probe sequences, excluding the terminal polymorphic position, to the mouse reference genome (mm10/GRCm38 build) with bwa mem v0.7.12 (Li 2013) using default parameters. The annotated position for a marker is the 1+(coordinate of the $3^{\prime }$ aligned end of the probe sequence), on the aligned strand. For probes that align equally well to multiple positions, a position was chosen at random. Markers whose probe sequence did not align to the reference genome were assigned a missing value for chromosome and a position of 0. Markers coincident with known SNPs from the Sanger Mouse Genomes Project were identified using bedtools intersect v2.22.1 (Quinlan and Hall 2010) and annotated with an rsID if available.

### Reference samples

A diverse panel of 522 samples was chosen for calibrating and evaluating the performance of the array. These included 49 classical laboratory strains, 12 wild-derived strains, 53 ${\text{F}}_{1}$ hybrids between inbred strains, 62 ${\text{F}}_{1}$ hybrids between lines from the CC, 100 individuals from the DO, 29 wild-caught *M. musculus* specimens, and 20 specimens from other *Mus* species. Because the array was designed to be maximally informative in the CC and DO, we included in our reference panel eight technical replicates (corresponding to at least three biological replicates) for each of the eight founder strains of the CC. All reference samples are listed in Supporting Information, Table S1.

The method of DNA preparation is indicated in Table S1. DNA stocks for most classical inbred strains were purchased from the Jackson Laboratory (“Jax”). High-molecular-weight DNA (“HMW”) from most ${\text{F}}_{1}$ hybrids and wild-caught specimens was extracted from tissues using a standard phenol-chloroform method (Sambrook and Russell 2006). DNA from most other samples was prepared from tail clips or spleens using the Qiagen DNeasy Blood & Tissue Kit (catalog no. 69506; Qiagen, Hilden, Germany) (“Qiagen”). DNAs donated by other laboratories are listed as “external.”

Samples indicated as “SGCF” in Table S1 were processed by the UNC Systems Genetics Core Facility. The UNC SGCF service includes DNA extraction from tissue samples; preparation of DNAs for shipment to Neogen Inc.; data processing and storage; and consultation on interpretation of genotype data.

### Array hybridization and genotype calling

Approximately 1.5  μg  genomic DNA per sample was shipped to Neogen Inc. (Lincoln, NE) for array hybridization. Genotypes were called jointly for all reference samples using the GenCall algorithm implemented in the Illumina BeadStudio software.

### Quality control

Arrays were subject to three quality checks before further analysis: (1) distribution of total hybridization intensity; (2) total number of missing and heterozygous calls; and (3) concordance between known sex of each sample and calls on the sex chromosomes.

#### (1) Hybridization intensity:

**Figure 1:**
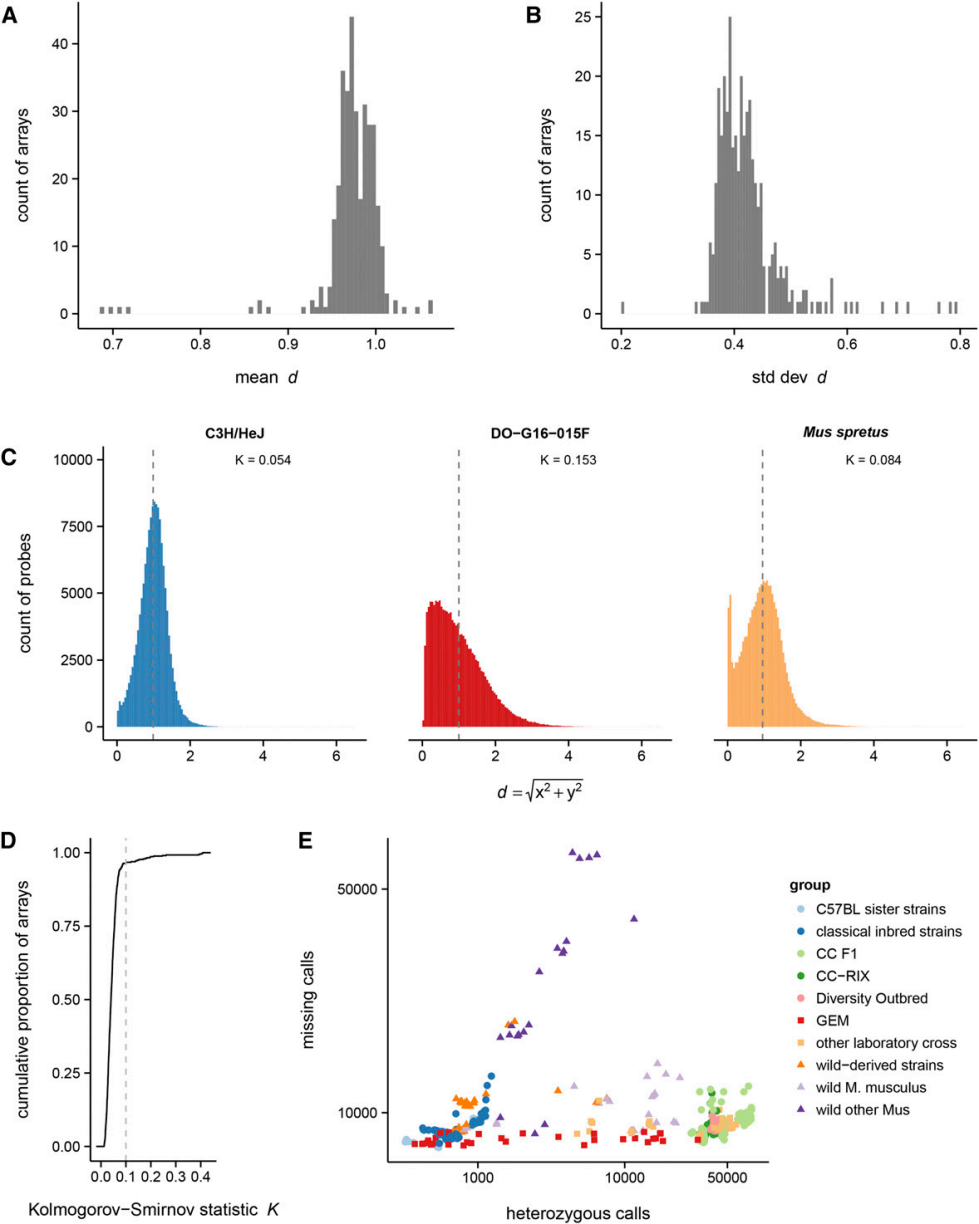
Quality checks for GigaMUGA arrays. Distribution of per-array mean (A) and standard deviation (B) of total hybridization intensity *d* (see *Materials and Methods*). (C) Examples of the distribution of *d* within single arrays. From left to right: a high-quality array (an inbred C3H/HeJ mouse), with approximately symmetric distribution and mean near 1.0; a failed array (a Diversity Outbred mouse), with right-skewed distribution; and a high-quality array for a genetically divergent individual (species *Mus spretus*), whose distribution is a mixture of a symmetric component and a spike near zero. (D) Cumulative distribution of Kolmogorov-Smirnov statistic (*K*) for departure from the expected N(0.97,0.42) distribution of *d*. (E) Count of missing calls *vs.* heterozygous calls for reference samples, by sample group (see Table S1).

Let $x_{0}$ and $y_{0}$ be the raw hybridization intensity values for the reference and alternate alleles, respectively, within a hybridization batch. Illumina’s normalization procedure transforms $x_{0}\to x$ and $y_{0}\to y$ such that x+y≈1 and the two homozygous clusters lay along the axes of a two-dimensional coordinate plane (Peiffer *et al.* 2006). Our group has anecdotally observed that, within an array, $d=\sqrt{x+y}$ is a slightly better measure of total intensity than R=x+y. (*R* overestimates intensity in highly heterozygous samples because, by the triangle inequality, |x+y|≤|x|+|y|.) The distribution of within-array mean and standard deviation of *d* across 522 arrays is shown in Figure 1, A and B.

The distribution of *d* within an array is an important indicator of genotyping quality. We recognize three general patterns (Figure 1C). For successful arrays (left panel), *d* has an approximately symmetric distribution, with mean 0.97 and standard deviation 0.42. A distribution of *d* skewed toward low values (middle panel) is associated with a high proportion of missing genotype calls, and indicates a failed array (Didion *et al.* 2014). Finally, the distribution of *d* for samples that are diverged from the mouse reference genome (right panel) is a mixture of a symmetric distribution, with mean near 1, and a spike near 0. This spike represents a population of probes whose hybridization is disrupted by off-target variants within the probe sequence (Didion *et al.* 2012).

Based on these observations, we computed the Kolmogorov-Smirnov statistic *K* for difference in the distribution of *d* from N(0.97,0.42) for each sample and flagged 18 samples at an empirically defined threshold of K>0.1 (Figure 1D).

#### (2) Call rate:

We inspected the rate of missing and heterozygous calls within groups of reference samples to establish group-specific thresholds (Figure 1E and Figure S1). A set of 12 *Mus musculus* samples with >15,000 missing calls, and samples of other *Mus* species with >45,000 missing calls, were flagged. An additional four samples from classical inbred strains with >2000 heterozygous calls were flagged.

#### (3) Concordance for sex chromosomes:

Female samples should have zero nonmissing calls at truly Y-linked markers, while males should be hemizygous. We counted the number of nonmissing, nonheterozygous calls at markers nominally mapped to the Y chromosome among samples known to be female (27±2.9, median ± MAD; maximum 33), or male (51±2.9; minimum 42). Four samples fell in the ambiguous range (more than 33 but less than 42 good calls at Y-chromosome markers), but all were from other Mus species. We computed the mean value of *d* (total intensity) at probes on the X chromosome within each sample as an additional check for sex-chromosome concordance. Female samples should have higher hybridization intensity on the X since they have two copies. On the basis of X-chromosome intensity, the four ambiguous samples were confirmed to be male. A visual summary of the sex-chromosome analyses is provided in Figure S2.

In total, 22 samples failed one or more quality filters (marked as “FAIL” in Table S1), leaving a final set of 500 reference samples (marked as “PASS”), which was used in subsequent analyses.

### Normalization

We transformed x,y to sum intensity R=x+y, and angle θ=2πarctan(x/y). (As noted above, *d* is a slightly better estimate of total intensity that *R*, but we use *R* for consistency with published methods.) We then computed the log2(intensity ratio) (LRR) and B-allele frequency (BAF) transformations defined in Peiffer *et al.* (2006) using a modified form of the Illumina-specific thresholded quantile normalization (tQN) approach proposed by Staaf *et al.* (2008). These normalization procedures require precomputed centroids for each of the three canonical genotype clusters (AA, AB, BB) at each marker. We estimated these centroids as the trimmed mean (omitting the most extreme 5% of values) of *R* and θ among samples called AA, AB or BB at each marker.

### Identification of multiallelic probes

The number of clusters (in the x,y-plane) for each probe was determined using a nonparametric method that leverages parent–offspring trios (Kao *et al.* 2014). Briefly, the algorithm proceeds in two steps: first, samples from the eight founder strains of the CC are used to identify clusters representing homozygous states. These clusters are iteratively merged using a *k*-nearest-neighbor approach. Second, samples from each of the $\left(\begin{array}{c} 8\\ 2 \end{array}\right)=28$ possible ${\text{F}}_{1}$ genotypes are assigned either to a new cluster or to an existing cluster, depending on the cluster assignment of their respective parents. The *k*-nearest-neighbor merging procedure is repeated to yield a final set of clusters for each marker.

### Phylogenetic analyses

We assessed the phylogenetic information content of GigaMUGA on the male-specific portion of the Y chromosome and the mitochondrial genome. These sequences are commonly used for phylogenetic analyses because they are both hemizygous and nonrecombining, and because each provides complementary insight into ancestry and demographic history. A set of 67 male samples (Table S1) was selected to span the three principal subspecies of *M. musculus*, including wild, wild-derived, and classical laboratory mice, plus the outgroup species *Mus spretus*. Genotype calls at 83 Y-chromosome markers, and 32 mitochondrial markers, were recoded to capture information from probes with aberrant hybridization patterns due to off-target variation in or near the probe sequence [“variable-intensity oligonucleotides”, or VINOs; Didion *et al.* (2012)]. At each marker, heterozygous calls and no-calls were assigned random nonallelic nucleotides: for instance, at a [T/G] SNP, a heterozygous call might be assigned A, and no-call might be assigned C. A parsimony tree was inferred separately for the resulting Y-chromosome and mitochondrial genotype matrices with RAxML v8.1.9 (Stamatakis 2014). Although the topology of these trees is likely to be meaningful, branch lengths are distorted by ascertainment bias in the SNP panel. The trees in Figure 7 are plotted with uniform branch lengths.

### Inspection of B6.PL-*Thy1^a^*/CyJ congenic line

The genetic background of the B6.PL-*Thy1^a^*/CyJ line (JAX stock number 000406) was investigated using a single male sample. This line carries a *Thy1* allele from PL/J in a C57BL/6 background. We used a HMM to reconstruct that sample’s genome as a mosaic of contributions from C57BL/6J, C57BL/6NJ, C57BL/6CR (Charles River), C57BL/6Tc (Taconic), C57BL/10ScN, and NON/ShiLtJ. The PL/J strain was not included in our set of reference samples, so we chose NON/ShiLtJ as a surrogate because it shares most of the interval around *Thy1* identical-by-descent with PL/J (Yang *et al.* 2011). We found that, although the HMM could easily identify contributions from non-J substrains of C57BL/6, it could not robustly discriminate between the several non-J substrains (owing to the paucity of informative markers in these comparisons, Table 4). Intervals consistent with C57BL/6 ancestry for which C57BL/6J can be ruled out as the donor were therefore simply labeled “non-C57BL/6J.”

### Performance of probes for genetically engineered constructs

To test the performance of the assays tracking the presence of genetically engineered constructs, we used 587 mouse samples that have been genotyped on the MegaMUGA platform, representing both samples known or presumed to carry at least one of the constructs and samples known to be devoid of them. Cluster plots of the raw *x*- and *y*-intensities for all 83 constructed-targeted probes on MegaMUGA were manually inspected. A subset of 38 was designated as informative on the basis of clustering patterns: samples known or presumed to carry the targeted construct had relatively high raw intensity on the expected axis (the allele corresponding to the true sequence of the construct), while negative control samples had low raw intensity. The 38 markers were grouped according to the targeted construct. Within each target, raw intensity (again, along only the informative axis) was summed across probes and a two-component (absence *vs.* presence) Gaussian mixture model was fit to the log10 sum intensities using the R package mclust (Fraley *et al.* 2012). A table of probe IDs, targets and informative alleles is provided in Table S3.

### Data availability

Genotype calls and hybridization intensity data (both raw and processed) for 522 reference samples are available for download from http://csbio.unc.edu/MUGA. Routines for quality checks and intensity normalization are implemented in the R package argyle, described elsewhere (Morgan 2016, this issue), and available for download from GitHub (https://github.com/andrewparkermorgan/argyle).

The GigaMUGA genotyping service is provided exclusively by Neogen Inc., Lincoln, NE. Users may provide samples as tissues or DNA aliquots. Data are returned in Illumina BeadStudio format via a secure file transfer. The University of North Carolina Systems Genetics Core Facility offers sample preparation, shipment to Neogen, and postprocessing of data to both internal and external users.

Annotation files for the MUGA family of arrays are available from http://csbio.unc.edu/MUGA.

## Results and Discussion

The final GigaMUGA array comprises 143,259 probes distributed across all 19 mouse autosomes, the X- and Y-chromosomes, and the mitochondrial genome. Of these, 67,645 (47.2%) were carried over from MegaMUGA. The vast majority of probes (141,090; 98.5%) are designed to interrogate biallelic SNPs, with the remainder designed to assay copy number (2006; 1.4%), multiallelic loci (34;0.02%), or the presence of engineered constructs (129;  0.01%). We classified probes into nine types (Table 1) based on the types of variants they target, and how they were ascertained.

**Table 1:** Probe types on GigaMUGA

Probe Type	Number	Description
Haplotype discrimination	54,250	SNPs selected for maximal information content with respect to CC/DO founders; called by Sanger Mouse Genomes Project or lifted over from Mouse Diversity Array (MDA) (Yang *et al.* 2009)
Recombination hotspot	46,020	Same as above, but selected to flank a catalog of 25,000 recombination hotspots from Baker *et al.* (2015)
Wild alleles	20,237	SNPs predicted to be segregating in wild mice, from MDA and whole-genome sequencing of wild mice
Other existing	13,036	Other SNP probes carried over from MDA
ICR novel	3693	SNPs segregating within or between selection lines derived from the ICR:Hsd outbred stock, ascertained from whole-genome sequencing
CNV/SD	2006	Non-SNP probes targeted at segmentally duplicated regions, intended for exploring CNV
Sister strains	1744	SNPs segregating between closely related inbred strains
Target locus	201	Probes targeting specific endogenous loci (*Xce*, *Vkorc1*, *R2d2*, genes in the complement cascade); most are not designed as SNP probes
Transgene	129	Presence-absence probes for detection of exogenous engineered constructs

**Figure 2:**
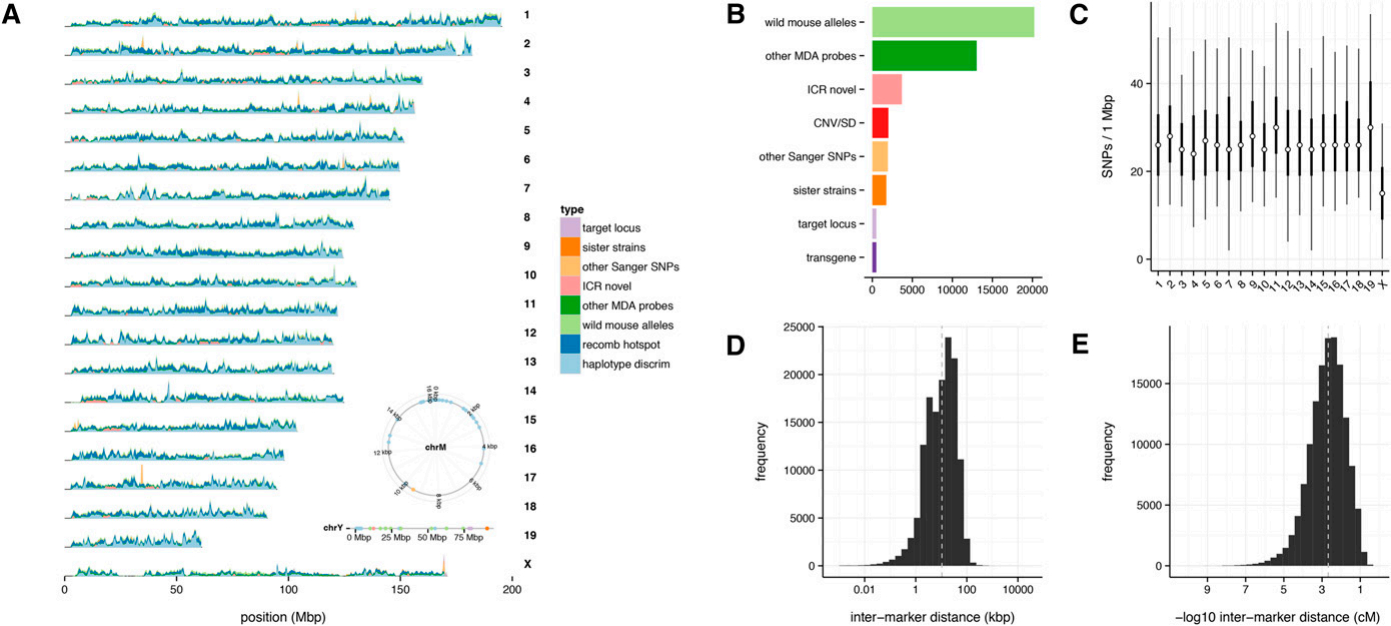
Genomic distribution of GigaMUGA probes. (A) Marker density across the autosomes and X-chromosome, plotted in 500-kb bins. Fill color indicates probe type. Individual markers are shown for the Y-chromosome and mitochondrial genome in the insets at right. (B) Relative representation of markers in all but the largest two classes (“haplotype discrimination” and “recombination hotspot”). (C) Distribution of marker density, in markers per Mb, for probes targeting biallelic SNPs (see *Materials and Methods* ). Dot indicates median, dark bar $25^{\text{th}}-75^{\text{th}}$ percentile, light bar $10^{\text{th}}-90^{\text{th}}$ percentile. (D) Distribution of physical distance between adjacent marker pairs. (E) Distribution of genetic distance between adjacent marker pairs, calculated by linear interpolation on the genetic map of Liu *et al.* (2014).

The genomic distribution of SNP and copy-number probes is shown in Figure 2. SNP probes are tiled along the autosomes every 10.4±12.1 kb (median ± 1 median absolute deviation) or every 0.002±0.003 cM, and every 16.1±20.2 kb (0.003±0.004 cM) on the X-chromosome. The nonrecombining Y-chromosome and mitochondrial genome are tagged with 83 and 32 probes, respectively. Because recombination is enriched in subtelomeric regions in mouse (Liu *et al.* 2014), the density of probes is higher at the distal ends of the autosomes than at the proximal ends. A final annotated array manifest is available in Table S2. The performance of GigaMUGA was assessed in a panel of 522 reference samples, of which 500 passed quality controls. All reference samples are listed in Table S1.

### Assignment of probes to quality tiers

Probes were assigned to four (mutually exclusive) tiers of decreasing quality based on their performance as biallelic SNP markers in the set of reference samples, using the following criteria. We denote genotype calls as “AA”, homozygous for the reference (C57BL/6J) allele; “BB”, homozygous for the alternate allele; “AB”, heterozygous; and “N”, no-call (missing).

- Tier 1:≥1 sample called each of AA, BB and AB, with no-call rate <10%
- Tier 2: all probes not in Tier 1, with ≥1 sample called each of AA and BB, with no-call rate <10%
- Tier 3: all probes not in Tiers 1 or 2, with no-call rate <10%
- Tier 4: all remaining probes

These definitions are motivated by the observation that the Illumina intensity-normalization and genotype-calling algorithms perform best when all three genotype states (AA, BB, AB) are present for each probe. However, assignment of probes to quality tiers is dependent on the composition of the set of reference samples: markers with low expected minor-allele frequency are unlikely to be represented in both homozygous states. Because both the content of the array, and the composition of the reference sample set are biased toward genetic backgrounds represented in common laboratory strains and the CC, quality tiers are particularly relevant to users of those and other common laboratory mouse strains. Users applying the array in other populations, such as wild-caught mice, should verify that probes perform as expected in their populations of interest. (We note that probes in lower quality tiers still provide information if treated as multiallelic markers and/or copy-number probes.) Assignments are summarized in Table 2.

**Table 2:** Allocation of probes to quality tiers

Probe Type \ Quality Tier	1	2	3	4
Haplotype discrimination	48,421	34	2033	3762
Recombination hotspot	39,757	53	1429	4781
Wild alleles	15,343	298	2553	2043
Other existing	12,133	380	292	231
ICR novel	1608	53	1120	912
CNV/SD	70	32	1650	254
Sanger known	1578	15	108	242
Sister strains	987	484	142	131
Target locus	50	3	63	85
Transgene	51	0	9	69
Total	119,998	1352	9399	12,510

### Genotype call rate and concordance between replicates

**Figure 3:**
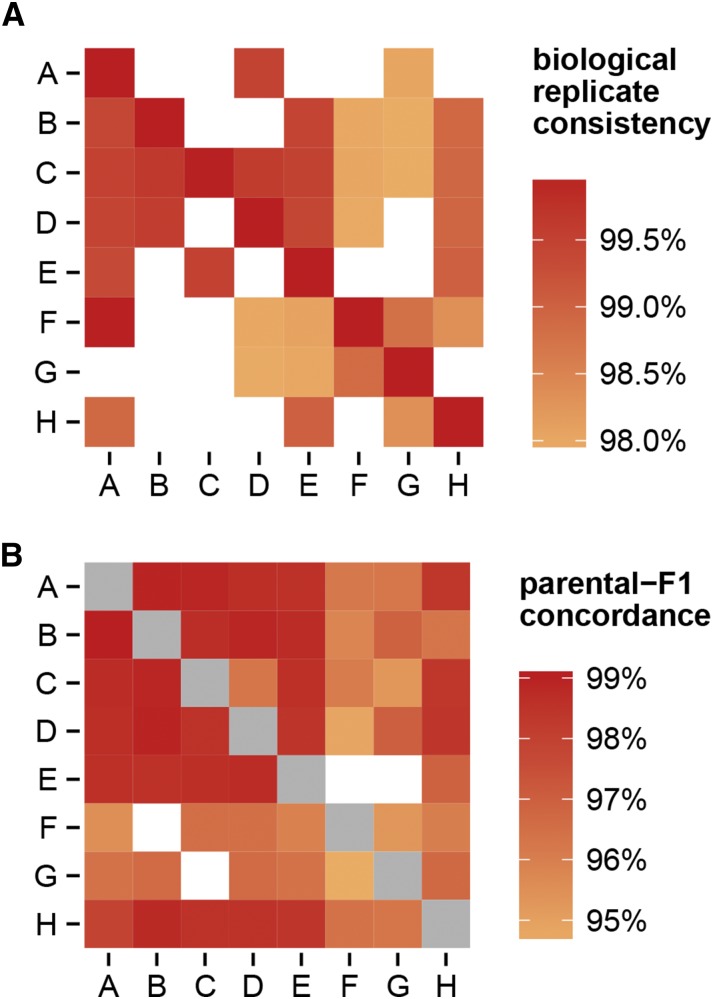
Concordance in genotype calls. (A) Concordance between biological replicates of the eight founder strains of the Collaborative Cross (on the diagonal), and ${\text{F}}_{1}$ hybrids between them (off the diagonal.) Maternal strain is indicated on the vertical axis, and paternal strain on the horizontal axis. Strain names are abbreviated as: A, A/J; B, C57BL/6J; C, 129S1/SvImJ; D, NOD/ShiLtJ; E, NZO/HlLtJ; F, CAST/EiJ; G, PWK/PhJ; H, WSB/EiJ. Blank cells indicate missing ${\text{F}}_{1}$ combinations. Note that the E×F and E×G crosses do not produce viable offspring. (B) Concordance between observed genotypes in ${\text{F}}_{1}$ hybrids and predicted genotype based on genotypes of the parental strains. Note the difference in color scale between panels. Gray cells indicate homozygous genotypes which are omitted from this analysis.

Among probes in tiers 1–3, the rate of nonmissing genotype calls is 99.99%±0.03% (mean ± standard deviation). The rate of concordance between 33 biological replicates of inbred strains is 99.99%±0.01%, and 98.84%±0.67% in 79 biological replicates of ${\text{F}}_{1}$ hybrids (Figure 3). Concordance between the observed autosomal genotypes in ${\text{F}}_{1}$ hybrids and the predicted genotypes based on parental strains is somewhat lower at 96.5%±6.9%. This decrease is due almost entirely to ${\text{F}}_{1}$s for which one parent is a wild-derived strain (Figure 3), and is therefore likely attributable to off-target variation in or near probe sequences in those strains (VINOs). VINOs are especially difficult to genotype in the heterozygous state (Didion *et al.* 2012).

### Multiallelic probes

**Figure 4:**
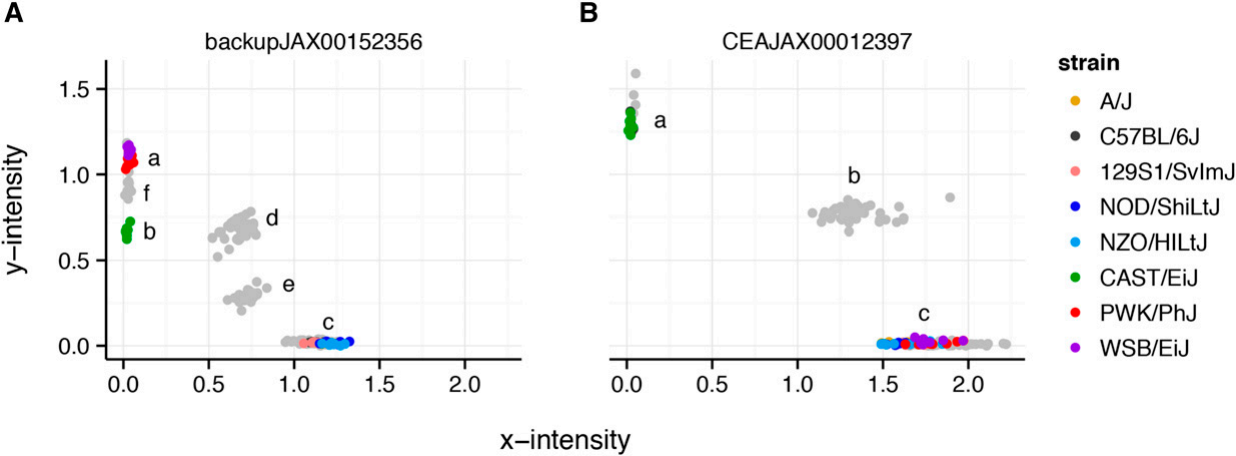
Hybridization patterns at a multiallelic probe. (A) A probe with six distinct genotype clusters. Each point represents one sample; colored points are the Collaborative Cross founder strains and gray points are ${\text{F}}_{1}$s between them. Although the standard calling algorithm would assign WSB/EiJ, PWK/PhJ and CAST/EiJ, and their heterozygous combinations to the same genotype, it is clear that WSB/EiJ and PWK/PhJ (cluster a) have a different allele than CAST/EiJ (cluster b). The remaining five strains form a single homozygous cluster (cluster c). Cluster d corresponds to the heterozygotes between clusters a and c, and cluster f the heterozygotes between clusters a and b. (B) For comparison, a probe that behaves as a standard biallelic SNP marker with three clusters, two homozygous (clusters a, c), and one heterozygous (cluster b).

Although most probes on the array were designed to behave as biallelic SNPs, we and others have observed that off-target variation in or near the probe sequence creates aberrant hybridization patterns that function as additional alleles or additional partially-informative markers (Didion *et al.* 2012). Distinguishing VINOs from sporadic no-calls requires a panel of training samples that includes replicates of all homozygous and heterozygous genotypes at each marker. Figure 4 shows an example of a standard biallelic probe with three clusters, and a mutiallelic probe with six clusters (representing three homozygous states and the corresponding three heterozygous combinations). We used a panel of 170 reference samples covering all 36 possible genotypes in the CC and DO to determine the number of clusters for each probe on GigaMUGA (Table 3). Although probes with three clusters in the CC—that is, probes that behave as biallelic SNPs—are the largest class among probe types designed to assay SNPs, additional alleles can be distinguished for 36,615 (27.0%). In the remainder of this report, we treat SNP probes as biallelic. Although this does not bias our results or interpretations of the overall utility of GigaMUGA, it does entail some loss of information (Fu *et al.* 2012).

**Table 3:** Number of alleles per probe, by probe type

Probe Type \ # Clusters	1	2	3	4	5	˃5
Haplotype discrimination	1699	765	37,146	8082	3232	1780
Recombination hotspot	1223	710	26,234	7240	4809	4334
Wild alleles	5329	1538	9151	1991	1015	432
Other existing	1710	286	7881	1109	613	218
ICR novel	1173	424	1045	352	251	376
CNV/SD	425	462	306	206	137	188
Sanger known	92	47	1182	247	155	116
Sister strains	670	163	561	94	75	52
Target locus	13	11	40	11	13	18
Transgene	1	1	36	6	6	2

### Information content in laboratory populations

**Figure 5:**
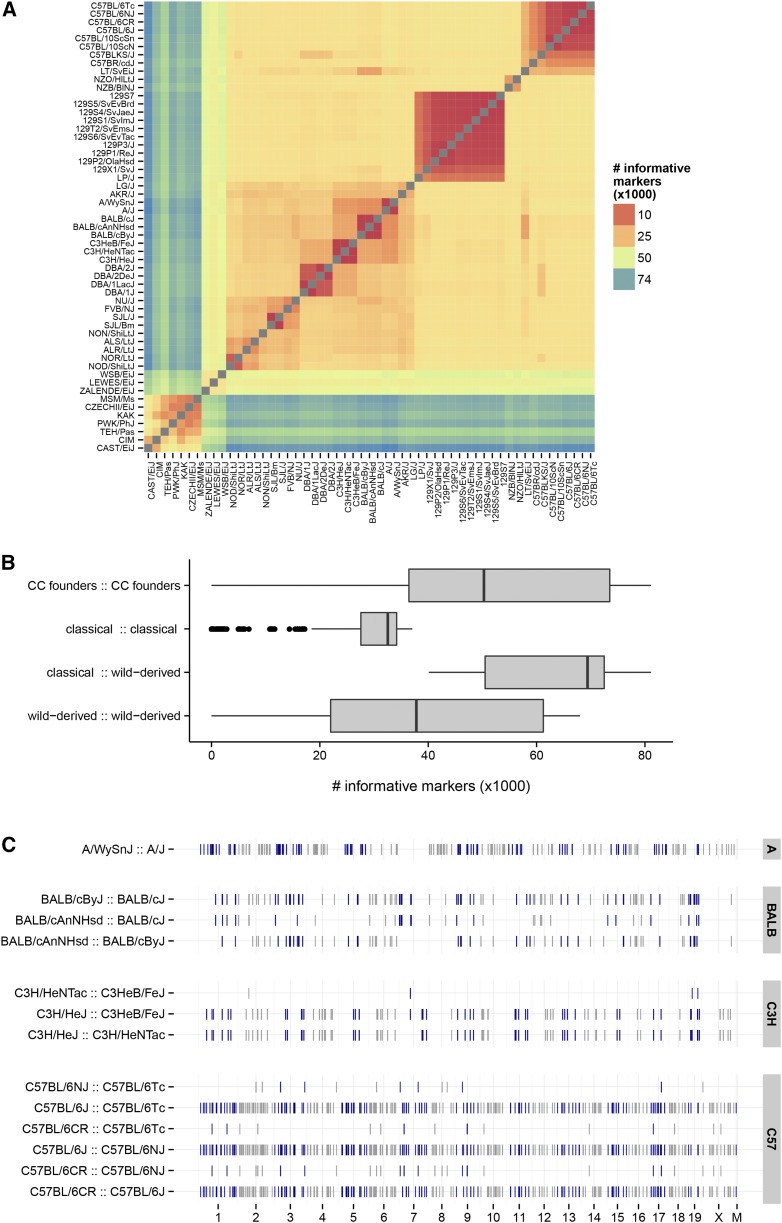
Pairwise informative markers between laboratory mouse strains. (A) Heatmap of the number of informative markers (SNP probes only; no special probes) between pairs of inbred strains. (B) Distribution of the number of informative markers among pairs chosen from different subsets of laboratory strains. (C) Markers informative in pairs of substrains. Each track shows the genomic position of markers informative between the two closely related inbred strains indicated at left. Points are colored as blue or gray on alternating chromosomes.

A key measure of the utility of a genotyping array for laboratory mice is the number of informative markers between commonly used inbred strains. We calculated the number of informative markers—markers in tiers 1 and 2 called for opposite homozygous genotypes in the members of a pair—between all pairs of 47 inbred strains (Figure 5). As expected, GigaMUGA is highly informative for the CC and DO, with a median of 50,285 markers expected to be segregating between any pair of CC founder strains. Although fewer markers (median 32,539) are informative between pairs of classical inbred strains, owing both to their shared ancestry and to our decisions about which SNPs to target, this number is still sufficient to achieve a density of ∼1 SNP per 100 kb.

An additional feature of GigaMUGA is its inclusion of probes for discriminating between substrains within several groups including the 129, BALB, C3H, C57BL/6, and DBA clusters. The genomic distribution of probes informative between selected substrains is shown in Figure 5, and corresponding counts in Table 4. For most substrain pairs, GigaMUGA provides markers on all autosomes, the X-chromosome, and the mitochondrial genome, at sufficient density to saturate the genome in a standard ${\text{F}}_{2}$ cross.

**Table 4:** Number of informative markers between closely related strains

	C57BL/6J	C57BL/6NJ	C57BL/6Tc						
C57BL/6CR	329	44	24						
C57BL/6J	⋅	373	351						
C57BL/6NJ	⋅	⋅	20						
	129P2/OlaHsd	129P3/J	129S1/SvImJ	129S4/SvJaeJ	129S5/SvEvBrd	129S6/SvEvTac	129S7	129T2/SvEmsJ	129X1/SvJ
129P1/ReJ	913	299	1521	1369	1326	2110	1301	1500	4620
129P2/OlaHsd	⋅	868	1982	1854	1807	2591	1771	2149	5232
129P3/J	⋅	⋅	1393	1234	1161	1971	1142	1595	4766
129S1/SvImJ	⋅	⋅	⋅	284	397	1247	391	774	5495
129S4/SvJaeJ	⋅	⋅	⋅	⋅	163	964	159	811	5539
129S5/SvEvBrd	⋅	⋅	⋅	⋅	⋅	913	2	856	5660
129S6/SvEvTac	⋅	⋅	⋅	⋅	⋅	⋅	880	1734	6444
129S7	⋅	⋅	⋅	⋅	⋅	⋅	⋅	843	5532
129T2/SvEmsJ	⋅	⋅	⋅	⋅	⋅	⋅	⋅	⋅	5186
	DBA/1LacJ	DBA/2J	DBA/2DeJ						
DBA/1J	76	4830	4594						
DBA/1LacJ	⋅	4760	4524						
DBA/2J	⋅	⋅	243						
	BALB/cByJ	BALB/cJ							
BALB/cAnNHsd	120	86							
BALB/cByJ	⋅	203							
	A/J								
A/WySnJ	310								
	C3H/HeNTac	C3HeB/FeJ							
C3H/HeJ	166	164							
C3H/HeNTac	⋅	5							
	SJL/J								
SJL/Bm	2								

**Figure 6:**
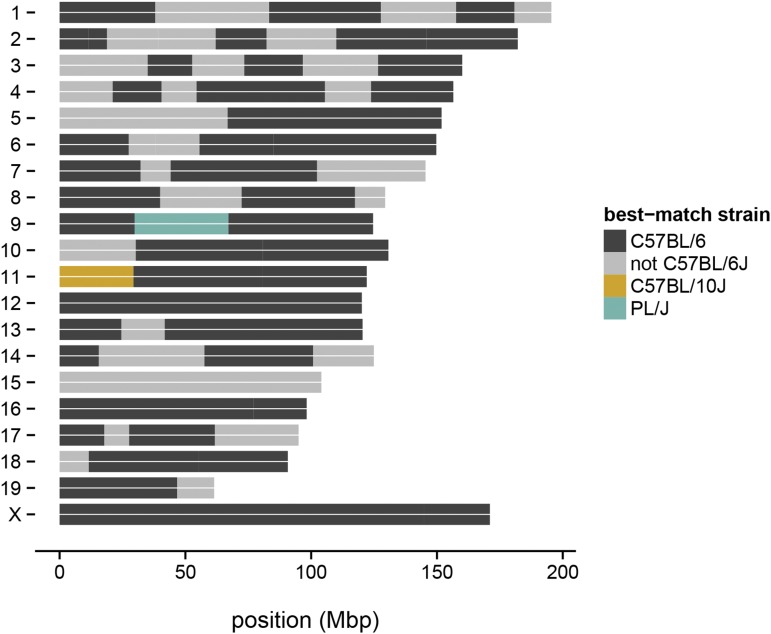
Verification of genetic backgrounds in a congenic line. Strain contributions to the congenic strain B6.PL-*Thy1^a^*/CyJ were reconstructed from genotype calls using a hidden Markov model. A large PL/J region (green) containing the *Thy1^a^* allele was identified on chromosome 9, as expected. Some contribution from C57BL/10J (gold) and C57BL/6 substrains besides C57BL/6J (gray) was also discovered.

The availability of informative markers between laboratory strains makes GigaMUGA a valuable tool for determining the components of the genetic background of laboratory stocks with substrain-level precision. Applications include verification of genetic background in knockout lines; precise characterization of congenic lines; and forensic examination of stocks or cell lines of unknown origin. As an example, we genotyped an individual from the B6.PL-*Thy1^a^*/CyJ strain (JAX stock number 000406). This congenic strain carries a *Thy1* allele from PL/J (at chr9: 44 Mb) backcrossed into a C57BL/6 background. We confirmed the presence of a large PL/J segment on chromosome 9 (Figure 6). Our analysis further identified contamination most likely from C57BL/10J on proximal chromosome 11, and suggests that one or more other substrains of C57BL/6 in addition to C57BL/6J contributed to the genetic background.

### Utility for population genetics and phylogeny

**Figure 7:**
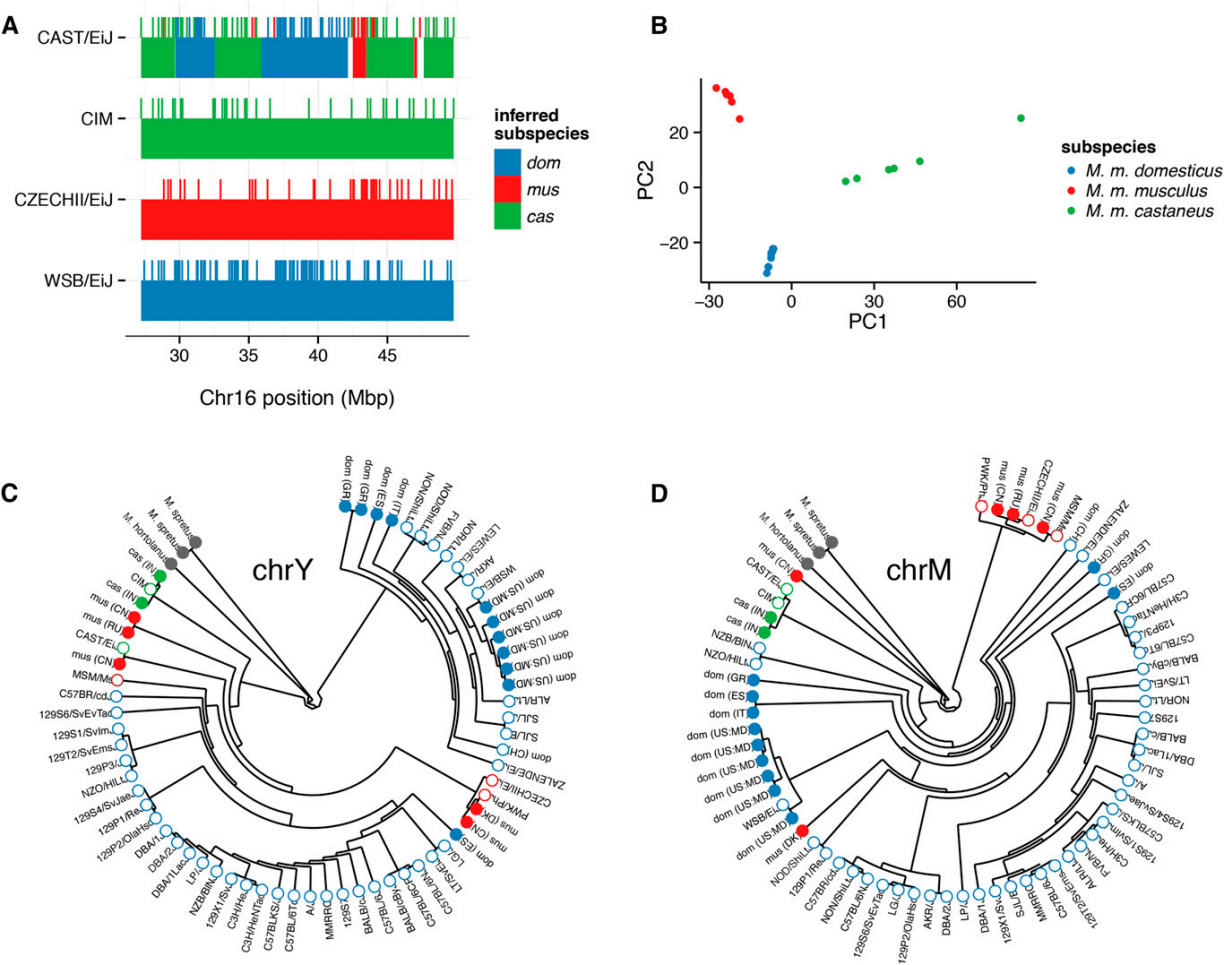
Phylogenetic information content of the GigaMUGA array. (A) Diagnostic alleles (blue, *M. m. domesticus*; red, *M. m. musculus*; green, *M. m. castaneus*) are shown as hash marks in a region of chromosome 16 where CAST/EiJ (top) has mixed ancestry. Pure representatives of each subspecies are shown for comparison. Smoothed ancestry blocks inferred using genotypes from the Mouse Diversity Array (Yang *et al.* 2011) are underlaid. (B) First two PCs from principal components analysis (PCA) of 20 wild *M. musculus* specimens at a randomly chosen subset of 3000 diagnostic markers (1000 per subspecies) on the autosomes. Individuals are colored according to their subspecies of origin, using the color scheme of panel A. (C) Phylogenetic tree constructed from 83 markers in the male-specific region of the Y chromosome in 67 male samples. Samples are colored according to their nominal subspecies or species of origin: blue, red and green as in panel A; maroon, *M. m. molossinus* (a *M. m. musculus* and *M. m. castaneus* hybrid); and gray, *Mus spretus*. See *Materials and Methods* for details of tree construction. Filled dots, wild-caught samples; open dots, inbred strains. (D) Phylogenetic tree for the same 67 male samples as in (C) but constructed from 32 mitochondrial markers.

We define a “diagnostic marker” as a marker at which genotype is informative for ancestry at the level of subspecies. Following the approach described in Yang *et al.* (2011) we used 30 wild-caught or wild-derived samples (19 *M. m. domesticus*, six *M. m. musculus* and five *M. m. castaneus*) with known pure ancestry and broad geographic distribution (Table S1) to identify 33,357 markers on the autosomes, X-chromosome, and mitochondrial genome at which the minor allele is present in only one subspecies. (We note that this definition is sensitive to the choice of reference samples, and to introgression: if any of the training samples carry an introgression tract, no diagnostic markers will be identified for the donor subspecies within that tract. We intend to revisit this problem with a more robust approach after more wild-caught training samples have been genotyped.) Because marker ascertainment was strongly biased toward SNPs segregating in *M. m. domesticus*, most diagnostic markers are diagnostic for *M. m. domesticus* (18,184), with fewer for *M. m. musculus* (7484) and *M. m. castaneus* (7689). Figure 7A demonstrates the ability of diagnostic SNPs on GigaMUGA to recover local ancestry in a region of chromosome 16 in which the CAST/EiJ strain was previously shown to have intersubspecific admixture (Yang *et al.* 2011).

To demonstrate the performance of GigaMUGA for phylogenetic studies in *M. musculus* and related species, we constructed trees using genotypes at 83 Y-chromosome probes and 32 mitochondrial probes. To mitigate ascertainment bias, we recoded genotypes as discrete characters based on clustering patterns (see *Materials and methods*) rather than using genotype calls directly. The resulting trees are shown in Figure 7, C–D. The Y-chromosome tree recovers known features of the patrilineal phylogeny of laboratory mice, including the presence of a *M. m. musculus* Y chromosome in most classical laboratory strains (Bishop *et al.* 1985), and in CAST/EiJ (Yang *et al.* 2011). The Y chromosome from wild pure *M. m. domesticus* constitutes a separate clade. The mitochondrial tree is concordant with the prior knowledge of the matrilineal phylogeny of house mice, separating the subspecies into monophyletic clades. It reveals evidence of intersubspecific hybridization in a wild sample trapped near the *musculus-domesticus* hybrid zone in Denmark (labeled “mus (DK)”): although most of its genome is of *M. m. musculus* origin, it has an *M. m. domesticus* mitochondrial genome.

### Copy-number analyses

Hybridization-intensity signals from Illumina arrays have two components informative for copy number: total intensity (*R*) and relative intensity from the alternative *vs.* the reference allele (*θ*). These can be normalized within and between arrays (Peiffer *et al.* 2006) to give the “log2-intensity ratio” (LRR) and “B-allele frequency” (BAF) respectively. Copy-number variants cause deviations of LRR away from zero and (at heterozygous sites) of BAF away from 0.5.

**Figure 8:**
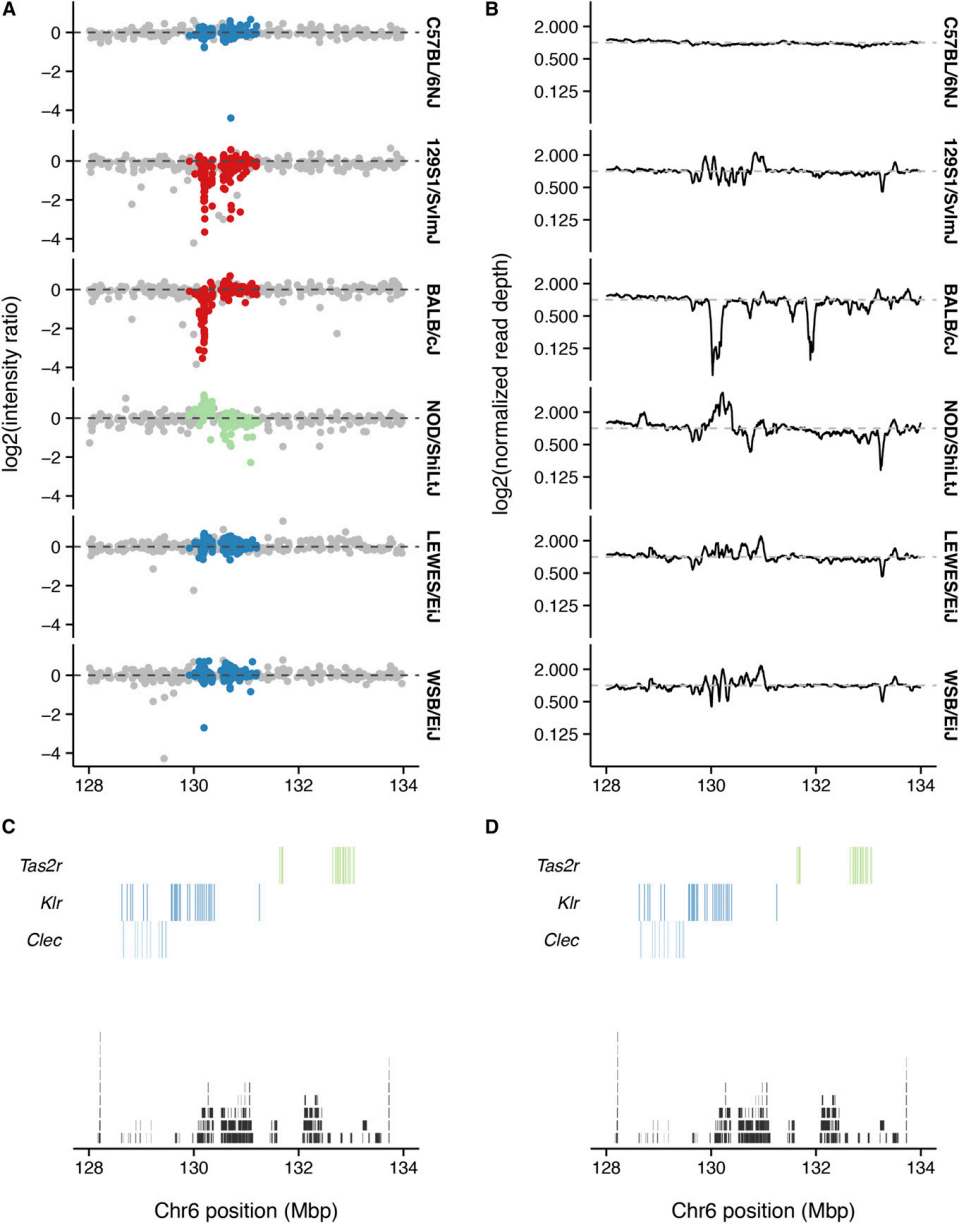
Detection of large copy-number variants. (A) Normalized hybridization intensity for several inbred strains at standard SNP probes (gray), or copy-number probes (colors) across the region chr6: 128−134 Mb. Strains with the reference copy number are shown in blue; those with putative deletions in red; and those with putative duplication in green. Dashed line indicates the reference value of zero. (B) Normalized read depth from whole-genome sequencing, calculated in 1-kb bins, for the same strains and region as in (A). Dashed line indicates the reference value of one. (C) Segmental duplications (black, from UCSC genomicSuperDups table) and genes from the *Clec*, *Klr* and *Tas2r* families (colors, from Ensembl). (D) Identical to (C), reproduced for reference against (B).

In addition to 141,090 SNP probes, GigaMUGA has 2006 copy-number probes, which are concentrated in segmentally duplicated regions of the mouse genome associated with recurrent structural mutations (Egan *et al.* 2007; She *et al.* 2008). To demonstrate the performance of GigaMUGA’s copy-number probes, we compared LRR and read depth from whole-genome sequencing in an interval on chromosome 6 (Figure 8) containing a known CNV (Keane *et al.* 2011). C57BL/6NJ, a close substrain of the C57BL/6J reference, has normal LRR and read depth. The BALB/cJ strain has reduced LRR across the targeted region, consistent with a deletion, while NOD/ShiLtJ has increased LRR, consistent with a duplication (Figure 8A). The wild-derived strains LEWES/EiJ and WSB/EiJ appear to have normal diploid copy number. Inspection of read-depth profiles (panel B) confirms a large deletion in BALB/cJ and a large duplication in NOD/ShiLtJ, with a more complex pattern of small gains and losses in 129S1/SvImJ, LEWES/EiJ and WSB/EiJ. The affected region is a patchwork of SDs, and contains genes from the *Klr* superfamily of immunoglobulin-like dendritic cell receptors.

Although optimization of CNV calling is beyond the scope of this manuscript, we note that existing software packages such as PennCNV (Wang *et al.* 2007) can make use of signal from both SNP probes and invariant copy-number probes on GigaMUGA.

### Targeted content: the complement cascade

The complement cascade bridges the innate and adaptive immune responses. Its constituent genes are well-defined, and functional polymorphisms within them underlie differential susceptibility to a variety of infectious and autoimmune diseases [see Beltrame *et al.* (2015) for a recent review]. Most of the genes within the complement cascade arose via ancestral gene duplications, and many are copy-number variable in mouse and human (Nonaka and Miyazawa 2001). We therefore designed 105 probes to directly genotype variants with functional significance within this important pathway, as well as further characterize copy number variation within the complement cascade across a range of mouse strains. They assay putative functional SNPs identified by the Sanger Mouse Genomes Project as segregating in the CC founder strains in 25 genes in the complement cascade (Table 5).

**Table 5:** Probes targeting functional variants in genes in the complement cascade

Gene symbol	Locus*^a^*	# Probes	Evidence for CNV?*^b^*
*Daf2*	1: 130.4	3	
*Cd55*	1: 130.4	4	
*Cd46*	1: 195.1	2	
*Serping1*	2: 84.8	1	19270705
*Cd59b*	2: 104.1	7	20308636, 21921910
*Cd59a*	2: 104.1	2	20308636
*Fstl5*	3: 76.4	1	
*Klhl32*	4: 24.7	1	
*C8a*	4: 104.9	2	
*C1qb*	4: 136.9	3	
*Masp2*	4: 148.6	4	
*Depdc5*	5: 32.9	1	
*Grm8*	6: 27.4	1	
*C1ra*	6: 124.5	6	
*C1s1*	6: 124.5	10	19270704, 19270705, 21921910, 17989247
*C1rb*	6: 124.6	3	19270705, 21921910
*C1s2*	6: 124.6	7	
*Cfd*	10: 79.9	2	19270705
*Pcdh9*	14: 93.2	1	
*C9*	15: 6.5	2	
*Masp1*	16: 23.5	2	
*C4b*	17: 34.7	16	21921910
*C4a*	17: 34.8	12	21921910, 17989247
*C2*	17: 34.9	1	
*C3*	17: 57.2	4	21921910

a Denoted as chromosome: position in Mb, in GRCm38/mm10 coordinates.

b Pubmed IDs of reports of CNVs >5 kb in size overlapping each locus. Key to references: 17989247, Cutler *et al.* (2007); 19270704, Cahan *et al.* (2009); 19270705, Henrichsen *et al.* (2009); 20308636, Quinlan *et al.* (2010); 21921910, Keane *et al.* (2011); 22916792, Wong *et al.* (2012).

**Figure 9:**
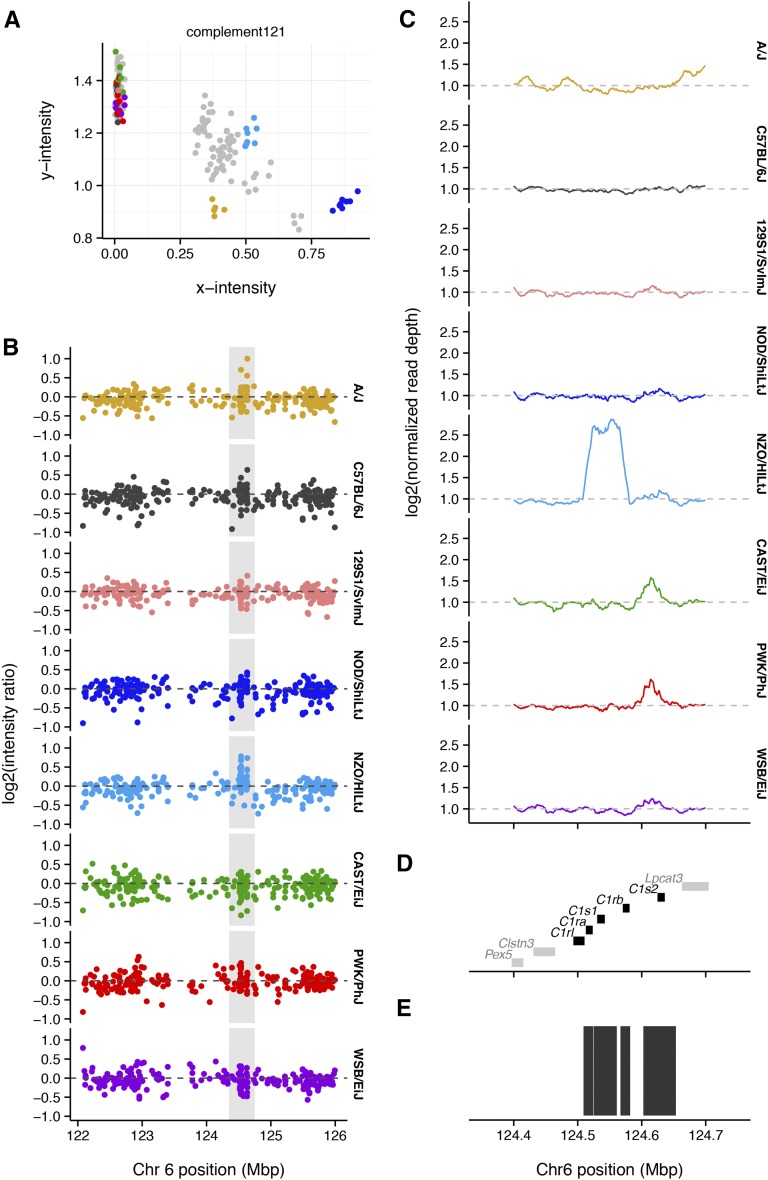
Probes targeted to complement-pathway genes detect copy-number variation. (A) Cluster plots for a probe in the *C1s1* gene. Inbred individuals from the CC founder strains are colored according to the scheme used throughout this paper; ${\text{F}}_{1}$s between them are colored gray. (B) Normalized hybridization intensity for one individual from each of the eight founder strains of the Collaborative Cross. Dashed line indicates the reference value of zero. (C) Normalized read depth from whole-genome sequencing, calculated in 500-bp bins, for the region highlighted in (A). Dashed line indicates the reference value of one. (D) Genes in the complement pathway (black) and other genes (gray), from Ensembl. (E) Segmental duplications (black, from UCSC genomicSuperDups table).

Interpretation of discrete genotypes calls at these probes is complicated by paralogy between genes in the complement pathway, and further by CNV: 25 of 105 complement probes (23.8%) have a no-call rate >10% compared to 6.4% array-wide. Most probes in these regions behave as multiallelic markers rather than biallelic SNPs (Figure 9A).

As an example, we focus on the genes encoding the C1 complex on chromosome 6. The C1 complex has two components, C1R and C1S, which arose by an ancient duplication near the base of the vertebrate lineage. Further duplications in the mouse lineage gave rise to *C1ra*, *C1rb*, *C1s1*, and *C1s2* (Nonaka and Miyazawa 2001). Hybridization patterns within *C1s1* (Figure 9, A and B) are characteristic of duplicated sequence. Apparently heterozygous calls in inbred strains—such as for NZO/HlLtJ at marker complement 120—are frequently diagnostic for cross-hybridization between paralogous sequences. In this case, both LRR and read depth from whole-genome sequence data indicate the presence of a large copy-number gain encompassing the entire *C1* region in NZO/HlLtJ (Figure 9, C–E). Its boundaries coincide with a segmental duplication in the reference genome. Integration of allele calls and intensity patterns at complement probes will be useful for directly characterizing alleles in the complement pathway.

### Targeted content: probes for engineered constructs

**Figure 10:**
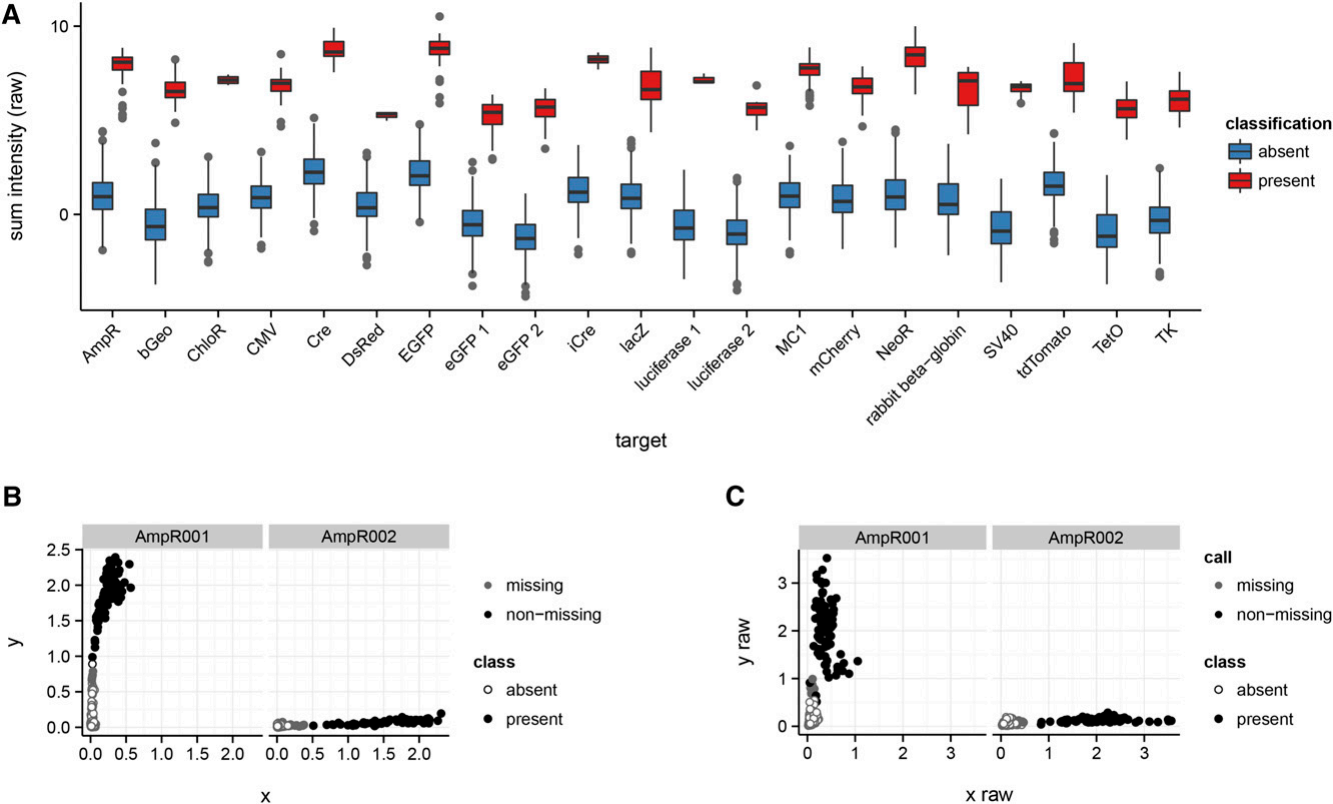
Performance of probe sets targeted to engineered constructs. (A) Distribution of sum-intensity among samples with (“present”, red), and without (“absent”, blue) each of 21 target constructs. Note that the scale for the *y*-axis is logarithmic. (B) Cluster plot for two probes targeted to an ampicillin resistance cassette (AmpR), showing normalized intensities. Points are colored according to the Illumina genotype call (N or H = missing; A or B = nonmissing), and shapes correspond to the classification inferred based on sum-intensity across all probes for this target. Note the curvilinear artifact for probe AmpR001. (C) Same as (B), but using raw intensities. Separation between positive and negative groups is slightly more obvious, and the curvilinear artifact is not present.

Probes targeted to engineered constructs were validated using raw intensity data from 587 samples genotyped on the MegaMUGA platform. A panel of 38 probes for 21 constructs provided robust discrimination between known negative and known or presumed positive samples (Figure 10). These probes are informative only for presence or absence, and do not discriminate between heterozygous or homozygous states. Furthermore, because only one allele at each probe exists (the other is arbitrarily chosen), the intensity normalization performed by Illumina BeadStudio introduces artifacts. We recommend using the raw fluorescence values for determining the presence or absence of engineered constructs.

### Concluding remarks

The Mouse Universal Genotyping Array (MUGA) series was designed to provide a low-cost, general-purpose solution for genotyping laboratory and wild mice. GigaMUGA array is the third generation of the MUGA platform. At 143,259 probes, it offers almost double the marker density of its predecessor, MegaMUGA, while retaining MegaMUGA’s top 85% best-performing markers. GigaMUGA’s content is optimized for discrimination between common laboratory strains, both classical and wild-derived, including substrains of very recent common origin. The array is also informative for ancestry and population structure in wild-caught and wild-derived mice. A new panel of copy-number probes tags regions of structural polymorphism to enable simultaneous CNV discovery and genotyping of SNPs.

Although the costs of sequencing continue to fall, analysis of sequencing datasets—especially from low-coverage or reduced-representation protocols (*e.g.*, RAD-seq)—remains challenging for nonexpert users. Furthermore, hybridization intensity even at biallelic SNPs can be used to detect copy-number variants. The robustness, simplicity and curated content of microarrays continues to make them a valuable tool in model organisms.

## Supplementary Material

Supporting Information
