# Supplementary material for: The Mouse Universal Genotyping Array: From Substrains to Subspecies
Source: G3 (Bethesda). 2015 Dec 18;6(2):263–79. doi: 10.1534/g3.115.022087 (PMC4751547; doi:10.1534/g3.115.022087)
Supplement: Supporting Information [file supp_6_2_263__index.html]

The Mouse Universal Genotyping Array: From Substrains to Subspecies — The Mouse Universal Genotyping Array: From Substrains to Subspecies — Supporting Information 

# The Mouse Universal Genotyping Array: From Substrains to Subspecies

## Supporting Information for Morgan *et al.*, 2016

**Files in this Data Supplement:**

- Figure S1 - Genotype call rate as a function of phylogenetic position within the *Mus* genus. (.pdf, 98 KB)
- Figure S2 - Use of call rates on the Y chromosome and X chromosome (A) and hybridization intensity on the X chromosome (B) to infer sex. (.pdf, 9 KB)
- Table S1 - Sample manifest: list of 522 reference samples genotyped with GigaMUGA, of which 500 pass quality control. (.xlsx, 36 KB)
- Table S2 - Array manifest: list of 143,259 probes on GigaMUGA. Positions are in build mm10/GRCm38 of the mouse reference genome. (.zip, 5872 KB)
- Table S3 - Presence/absence probes validated for detection of genetically-engineered constructs. (.xlsx, 11 KB)
