# Supplementary figures and images for: The Mouse Universal Genotyping Array: From Substrains to Subspecies
Source: G3 (Bethesda). 2015 Dec 18;6(2):263–79. doi: 10.1534/g3.115.022087 (PMC4751547; doi:10.1534/g3.115.022087)

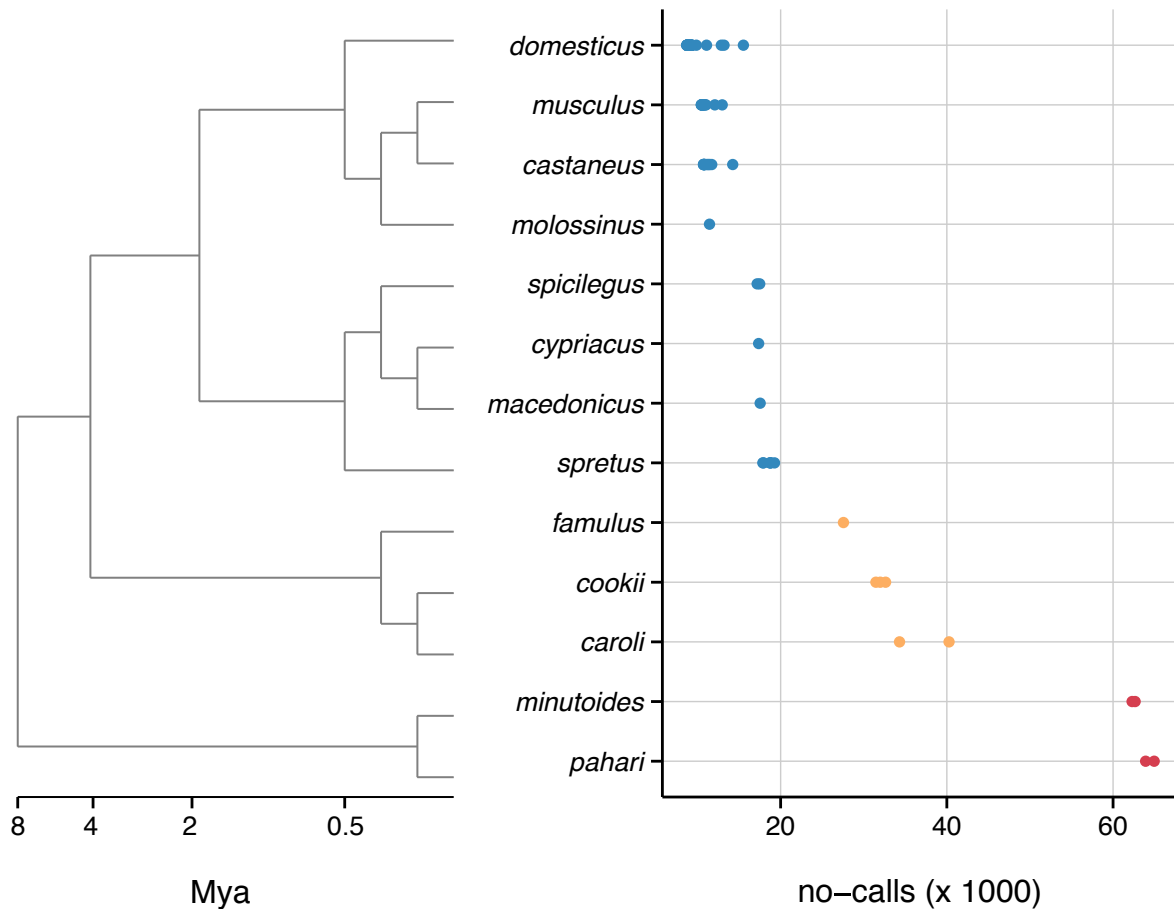

Supplement: Supporting Information [file supp_g3.115.022087_FigureS1.pdf]

**A**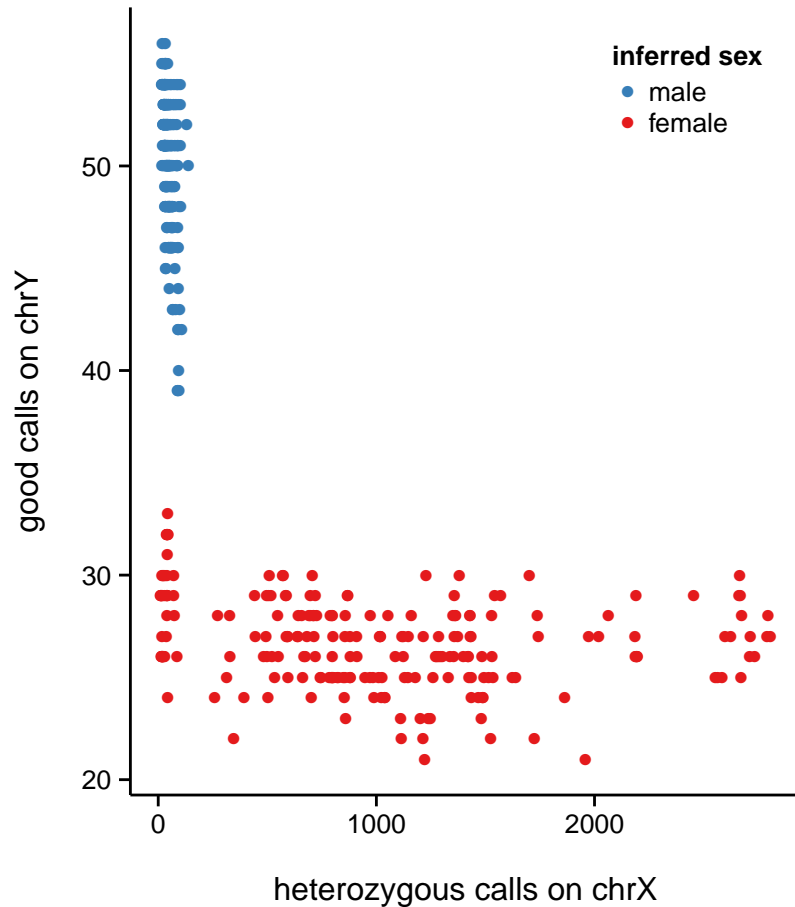**B**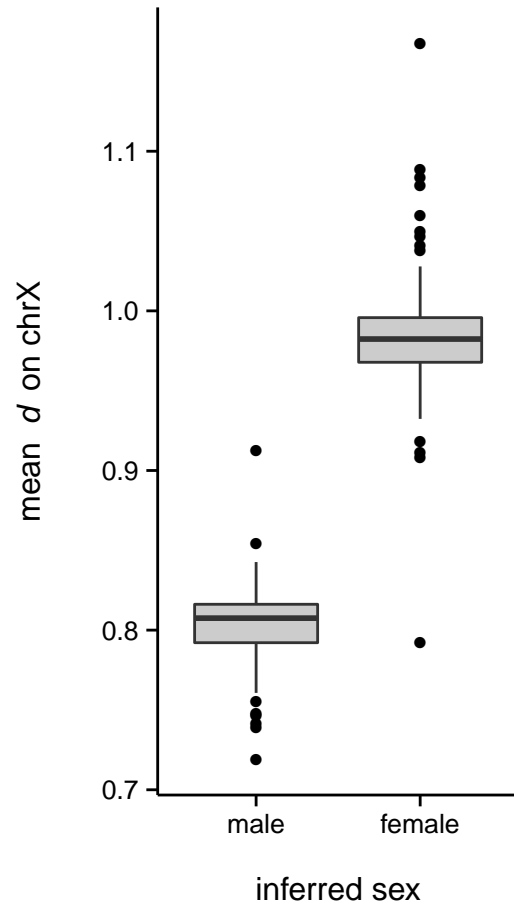

Supplement: Supporting Information [file supp_g3.115.022087_FigureS2.pdf]
